# Clinical efficacy of bronchoalveolar lavage in the treatment of small airway diseases in children

**DOI:** 10.3389/fped.2024.1373272

**Published:** 2024-05-09

**Authors:** Lili Zhou, Xuyun Peng, Liqin Cao, Lin Zhang, Huazheng Xiang

**Affiliations:** ^1^ Department of Pediatrics, Ganzhou Women and Children’ Hospital, Ganzhou, Jiangxi Province, China; ^2^ Department of Pediatrics, Shangyou Women and Children’s Hospital, Ganzhou, China; ^3^ Department of Pediatrics, Jian Women and Children’s Hospital, Jian, China

**Keywords:** bronchoalveolar lavage, small airway disease, children, HRCT, treatment

## Abstract

**Objective:**

This study aimed to evaluate the efficacy of bronchoalveolar lavage (BAL) in the treatment of children with small airway diseases.

**Methods:**

Children [*n* = 112; boys: 76, girls: 36 (ratio 2.1:1); age range: 1 month–10 years; median age: 12 months] with small airway diseases diagnosed by high-resolution computed tomography (HRCT) were enrolled in this study. The patients were assigned to either the BAL group (BAL and conventional therapy) or the control group (conventional therapy only). The duration of cough, fever, wheezing, hospitalization duration, disease course before admission, treatment cost, HRCT recovery time, and re-hospitalization rate were compared between the two groups.

**Results:**

The median disease course before admission of the BAL group patients was longer than that of the controls (*p* = 0.006). The duration of cough and wheezing in the BAL group was significantly longer than that in the control group (*p* = 0.012 and *p* = 0.001, respectively). The recovery time of cough, the re-hospitalization rate, and the total expenditure incurred for the BAL group were lower than those for the control group (*p* = 0.027, *p* = 0.026, and *p* = 0.000, respectively). At 2 months after discharge, the small airway lesions were found to be absorbed in 86.2% of BAL group patients vs. 64.1% of control group patients. At 6 months after discharge, the lesions were not fully absorbed in 3.4% of the BAL group patients compared to 20.5% in the control group patients.

**Conclusion:**

BAL is suitable for patients with a long disease course before admission, a long duration of coughing, and recurrent wheezing. BAL treatment of small airway diseases in children can promote the disappearance of clinical symptoms, accelerate the improvement of imaging, reduce the rate of re-hospitalization, and reduce the cost of treatment.

## Introduction

Small airway disease is a common group of children's respiratory system disease characterized by small airway obstruction. The small airway is relatively earlier and more easily invaded in respiratory system disease. The change of its function at the early stage of the disease, possibly induced by inflammation and mucus embolism, can be reversed. In the later stage, the small airway becomes fibrotic, deformed, narrow, or even closed, and its function becomes irreversible. The small airway disease is defined as a disease located beyond the seventh or eighth generation of the tracheobronchial tree with a diameter of <2 mm (1). The incidence of small airway diseases has been reported as 10%–20% by Berend (2). These include pneumonia (especially interstitial pneumonia), asthma, bronchiolitis, bronchiolitis obliterans, bronchiectasis, some congenital bronchopulmonary anomalies, and early disseminated tuberculosis. These diseases are difficult to diagnose early and treat. However, with the wide application of high-resolution computed tomography (HRCT) and pulmonary function, the detection rate and awareness about small airway diseases are increasing (3). Several small airway diseases are characterized by mucus accumulation, mucus embolism, plastic bronchitis, and uneven bronchial aeration, which cannot be relieved via conventional anti-infection treatment. Instead, bronchoalveolar lavage (BAL) therapy is required for such cases. For more than 40 years now, BAL has been widely used in pediatric respiratory diseases (4). Despite its frequent use, there is a lack of contemporary literature regarding the diagnostic utility of BAL for small airway diseases in children. BAL is a safe and minimally invasive treatment, but it is also complicated and involves certain risks that limit its clinical application. Therefore, we evaluated the utility of BAL in the treatment of small airway diseases in children in this study.

## Materials and methods

### Study population

#### Inclusion criteria

The inclusion criteria are as follows: (a) children with small airway diseases diagnosed by HRCT who were hospitalized for medical treatment at the Department of Pediatric Respiratory Medicine of our hospital from January 2021 to November 2022; (b) provision of the informed consent of the child or his parents; and (c) indications for bronchoalveolar lavage treatment and identification of pathogens.

#### Exclusion criteria

The exclusion criteria are as follows: (a) patients with contraindication of bronchoscopy; (b) patients with incomplete clinical data; and (c) patients with primary diseases such as those of the heart, brain, blood vessels, and hematopoietic system.

#### Diagnostic criteria

Small airway lesions are mainly bronchiolar lesions. The main HRCT features of small airway lesions are bronchial wall thickening, tree-in-bud sign, mosaic sign, and air trapping (5).

#### Study groups

Children with small airway diseases who had indications for BAL therapy were grouped according to their parents’ willingness to choose BAL therapy. Those who opted for BAL therapy were included in the BAL group and received BAL treatment along with conventional treatment; the control group included patients who were not willing to accept BAL treatment and received conventional treatment only. Based on their clinical symptoms, the children in the conventional treatments group were treated with anti-infection, oxygen inhalation, antipyretic, asthma relieving, atomization, phlegm reduction, sputum aspiration and elimination, nutrition, and so on.

#### Study contents

The duration of fever, cough, and wheezing; the recovery time of fever, cough, and wheezing; days of hospitalization; treatment cost; course of disease before admission; CT recovery time; re-hospitalization rate; and blood routine of the two groups were recorded. CT was performed at the time of hospital admission and then at 1, 2, 3, and 6 months after discharge. No more checks were performed after CT results returned to normal. Chest imaging was performed to examine whether the lesion showed no absorption, partial absorption, or complete absorption.

### Procedure for BAL

No contraindications for BAL were determined. Family members of the children signed informed consent forms. Preoperative routine examinations included blood routine tests, bleeding and clotting time records, and electrocardiograms. Patients were instructed to fast and abstain from water for 4–6 h before surgery. The patients were asked to inhale 2.5 mg of terbutaline atomizing solution 30 min before the surgery. Then, 5 mg of dexamethasone was injected to prevent laryngeal edema. The entire process was recorded using Japan Fujieneng EB-270S (outside diameter 4.9 mm) and Eb-270p (outside diameter 3.8 mm) pediatric electronic bronchoscopes, an EPX-2200 image processor, a universal light source, and an image display system. After anesthesia, the bronchoscope was inserted into the airway through the glottis, passing through the nasal cavity and throat. We observed the tracheal carina, each lobe, and segmental bronchus, as well as the lesions identified in the HRCT scan along the direction of the lens. Then, normal saline at 37°C was used for irrigation in stages using 0.50–1.00 ml/kg each time, and the inflammation or sputum supposition was brushed and rinsed according to the situation of the site. The douche solution was inhaled into a sterile container for inspection, and the intraoperative situation of the child was closely monitored. After the surgery, fasting and water prohibition were maintained for 2 h. We also looked for potential complications such as fever, hemoptysis, or dyspnea. The number of BAL treatments for each child was determined as deemed necessary.

### Ethical approval

The study protocol was approved by the Ethics and Research Council of Women and Children's Hospital of Ganzhou (2022-117) on 27 December 2022. The data were collected from the patients anonymously.

### Statistical analyses

The data were analyzed by using the SPSS 20.0 software package. Continuous variables were reported as the median (range) and compared using Student's *t*-test or the non-parametric Mann–Whitney *U*-test. The categorical variables were presented as numbers (%) and compared using the *χ*^2^ test. *p* < 0.05 was considered to indicate statistical significance.

## Results

### Demographic and clinical information

This study was retrospective. A total of 112 patients [boys: 76, girls: 36 (ratio 2.1:1); age range: 1 month–10 years; median age: 12 months] were enrolled. The re-hospitalization rate was 32 (28.6%). The pathogen positive rate was 74 (66.1%). The main viral infections were respiratory syncytial virus and rhinovirus. The main bacterial infections were *Streptococcus pneumoniae*, *Haemophilus influenzae*, and *Moraxella catarrhal*. The pathogens and the main discharge diagnosis are listed in Table 1.

**Table 1:** Demographic and clinical information of 112 patients.

Index	*N* = 112
Sex (male/female)	76/36
Age (months)	12 (1–120)
Re-hospitalization	32 (28.6%)
Pathogen positive	74 (66.1%)
Viral infection	15 (13.4%)
Bacterial infection	23 (20.5%)
*Mycoplasma pneumoniae* infection	10 (8.9%)
Single infection	48 (42.9%)
Co-infection	26 (23.2%)
Diagnosis	
Bronchiolitis	7 (6.3%)
Bacterial pneumonia	12 (10.7%)
Viral pneumonia	14 (12.5%)
*Mycoplasma pneumoniae*	10 (8.9%)
Chronic pneumonia	2 (1.8%)
Aspiration pneumonia	2 (1.8%)
Severe pneumonia	16 (14.3%)
Unclassified pneumonia	30 (26.8%)
Bronchopulmonary dysplasia	3 (2.7%)
Bronchiolitis obliterans	4 (3.6%)
Bronchial asthma	8 (7.2%)
Persistent bacterial bronchitis	1 (0.9%)
Bronchial foreign body	1 (0.9%)
Gastroesophageal reflux	1 (0.9%)

### Comparison between the BAL group and control group

The BAL group included 35 cases (boys:girls: 2.18:1; median age: 10 months), while the control group included 77 cases (boys:girls: 2.08:1; median age: 13 months). No significant difference was noted in terms of age, sex, hospitalization days, fever duration, and the recovery time of fever and wheezing between the two groups (*p* > 0.05). The total cost in the BAL group was shorter than that in the control group (*p* = 0.000). The median disease course before admission in the BAL group was longer than that in the control group (*p* = 0.006). The duration of coughing and wheezing in the BAL group was longer than that in the control group (*p* = 0.012 and *p* = 0.001, respectively). The re-hospitalization rate, the recovery time from coughing, and the total cost of treatment for the BAL group were significantly lower than those for the control group (*p* = 0.026, *p* = 0.027, and *p* = 0.000, respectively). No significant differences were noted in the levels of white blood cells, neutrophils, lymphocytes, hemoglobin, platelets, and C-reactive protein between the two groups (details are provided in Table 2).

**Table 2:** Comparison between BAL and control groups.

	BAL group (*n* = 35)	Control group (*n* = 77)	*p*
Sex (male)	24 (68.57%)	52 (67.53%)	0.55
Number of re-hospitalization times	5 (14.29%)	27 (35.06%)	0.026
Course of disease before admission (days)	30 (1–150)	14 (1–180)	0.006
Days of hospitalization	7 (2–14)	7 (2–26)	0.98
Age (months)	10 (2–120)	13 (1–80)	0.18
Duration of fever (days)	2 (0–9)	2 (0–17)	0.73
Recovery time of fever (days)	2 (0–6)	2 (1–13)	0.51
Duration of cough (days)	36 (0–159)	19 (0–183)	0.012
Recovery time of cough (days)	5 (1–13)	6 (1–19)	0.027
Duration of wheezing (days)	10 (0–93)	2 (0–72)	0.001
Recovery time of wheezing (days)	5 (1–14)	4 (1–23)	0.81
Total cost (yuan)	7,891 (4,653–16,387)	9,107 (1,998–58,809)	0.000
White blood cells at admission (×10^9^/L)	12.12 (4.77–28)	12.88 (4.26–21.9)	0.89
Neutrophils at admission (%)	34.4 (15–78.3)	42.1 (10–91.3)	0.071
Lymphocytes at admission (%)	58.8 (12–79.2)	44.4 (6.1–77.7)	0.067
Hemoglobin at admission (g/L)	126 (98–152)	124 (68–148)	0.60
Platelets at admission (×10^9^/L)	356 (201–667)	355 (7–606)	0.31
C-reactive protein (mg/L)	1.63 (0.1–40)	2 (0.1–51)	0.21

### Absorption of small airway lesions

Of the total, 68 children were followed up and re-examined with HRCT after discharge, including 29 in the BAL group and 39 in the control group. Details are provided in Table 3. The *χ*^2^ test revealed that *χ*^2 ^= 17.797, and *p* = 0.000 indicated a statistically significant difference at 1 month after discharge (see Table 4). The children with incomplete imaging absorption were reviewed 2 months after the discharge. The *χ*^2^ test showed that *χ*^2 ^= 20.179, and *p* = 0.000 indicated a statistically significant difference (see Table 5). Incomplete absorption after 6 months was noted in one (3.4%) case in the BAL group and eight (20.5%) cases in the control group.

**Table 3:** Recovery time of HRCT in BAL and control groups.

Group	Number	Completely absorbed				Partially absorbed
		1 month after discharge	2 months after discharge	3 months after discharge	6 months after discharge	More than 6 months after discharge
BAL group	29	1	24	1	2	1
Control group	39	6	19	5	1	8
Total	68	7	45	16	3	9

**Table 4:** Comparison of pulmonary CT absorption between the two groups at 1 month after discharge.

Group	Number	CT absorption of the lung at 1 month after discharge			
		Completely absorbed	Partially absorbed	Not absorbed	Absorption rate
BAL group	29	1	24	4	25 (86.2%)
Control group	39	6	21	12	27 (69.2%)
Total	68	7	45	16	52 (76.5%)
*χ* ^2^					17.797
*p*					0.000

**Table 5:** Comparison of pulmonary CT absorption between the two groups at 2 months after discharge.

Group	Number	CT absorption of lung at 2 months after discharge			
		Completely absorbed	Partially absorbed	Not absorbed	Absorption rate
BAL group	28	24	1	3	25 (89.3%)
Control group	33	19	5	9	24 (72.7%)
Total	61	43	6	12	49 (80.3%)
*χ* ^2^					20.179
*p*					0.000

## Discussion

The small airway disease cannot be satisfactorily treated by using the conventional treatment methods alone, and the chances of recurrence are high. In this study, fiberoptic bronchoscopy was used to perform BAL in children with small airway diseases. During the surgery, the lens directly reached the lesion site and removed the secretions and sputum suppositories in the small airways, which facilitated the rapid reduction of clinical symptoms. The study indicated that BAL was suitable for patients with long disease courses before admission, long durations of coughing, and recurrent wheezing episodes. It was reported that BAL treatment was effective in persistent bacterial bronchitis, which was a common cause of chronic wet cough in preschool children (6). It also confirmed that BAL could effectively reduce recurrent wheezing in young children (7). Several important factors, such as inflammatory factors, chemokines, cytology, and infectious microorganism etiology, can be analyzed by testing alveolar lavage fluid, which can be helpful for the diagnosis, observation, and prognosis of respiratory diseases (8). Based on our results, BAL did not reduce fever and wheezing time but shortened the coughing time. Minqing et al. reported that BAL treatment could effectively shorten the fever remission time and hospital stay duration and provide cough relief (9). The inconsistency in the literature may be attributed to the difference in the inclusion criteria. Currently, only a few pieces of literature analyze small airway diseases separately. Some patients with refractory pneumonia or severe pneumonia have small airway lesions (10). The present results showed that the re-hospitalization rate of the BAL group was significantly shorter than that of the control group. The literature on the re-hospitalization rate of BAL treatment is limited to date. The total cost incurred by the BAL group patients was significantly lower than that of the control group patients, which agrees with the research findings of Carr et al. (11). The present results showed that the small airway lesions were absorbed 2 months after discharge in the BAL group and after 6 months in the control group, indicating that BAL played a positive role in improving small airway lesions. Moreover, the recovery time of HRCT in the BAL group was significantly shorter than that in the control group. There are only a few literature reports on the imaging recovery time after alveolar lavage treatment.

In conclusion, BAL is a rapid and highly efficient treatment approach for small airway diseases in children. Our findings highlight the need to focus on small airway diseases in children, which is a common disease that is easily overlooked in clinics, and to provide a theoretical basis for its treatment.

However, this study is limited by the lack of lung function testing to evaluate the recovery of small airway diseases because the study patients were relatively young and could not independently complete the routine ventilation tests to fully reflect small airway lesions. Further research on lung functions would contribute to the complete understanding of the efficacy of the present treatment approach. This study included a relatively small number of children, and the children were from a unit of our hospital; thus, there may be some case-selective bias.

## Data Availability

The original contributions presented in the study are included in the article/Supplementary Material, further inquiries can be directed to the corresponding author.
